# Efficacy and safety of bronchial artery embolization in the treatment of hemoptysis:a systematic review and meta-analysis of RCTs

**DOI:** 10.3389/fmed.2026.1959450

**Published:** 2026-10-21

**Authors:** Minhui Zhang, Junchen Wang, Yunyun Shuai, Rongxuan Zhang, Xiaojuan Kou, Fan Wang, Zhaoxia Li, Haihong Li

**Affiliations:** 1 School of Nursing, Gansu University of Chinese Medicine, Lanzhou, Gansu, China; 2 Gansu Provincial Maternity and Child-care Hospital/Gansu Provincial Central Hospital, Lanzhou, Gansu, China; 3 The Second Hospital of Gansu Province, Lanzhou, Gansu, China; 4 School of Clinical Medicine, Northwest Minzu University, Lanzhou, Gansu, China; 5 The Second Hospital of Lanzhou, Lanzhou, Gansu, China

**Keywords:** bronchial artery embolization, hemoptysis, meta-analysis, randomized clinical trial, systematic review

## Abstract

**Background:**

Bronchial artery embolization (BAE) is widely used for hemoptysis, but the supporting evidence has mainly been derived from observational studies, and its certainty remains unclear. This study aimed to evaluate the efficacy and safety of BAE compared with other treatment modalities based exclusively on randomized controlled trials (RCTs), with evidence certainty assessed using the Grading of Recommendations Assessment, Development and Evaluation (GRADE) approach.

**Methods:**

We searched seven databases from inception to December 29, 2024, without language restrictions. Two reviewers independently screened studies, extracted data, and assessed risk of bias. Random-effects meta-analyses were performed, and evidence certainty was assessed using GRADE. Comparator-specific analyses were primary for the main outcomes, while pooled analyses across different comparators were exploratory. An updated search on June 24, 2026 identified no additional eligible RCTs. The review was registered in PROSPERO.

**Result:**

Twenty RCTs involving 1,594 patients were included. Compared with pharmacotherapy, BAE showed higher clinical efficacy (RR 1.24, 95% CI 1.18–1.31), lower recurrence (RR 0.26, 95% CI 0.13–0.54), lower mortality (RR 0.20, 95% CI 0.05–0.90), and fewer overall complications (RR 0.41, 95% CI 0.26–0.63), all supported by moderate-certainty evidence. BAE plus usual care versus usual care showed higher clinical efficacy (RR 1.27, 95% CI 1.12–1.43; very-low-certainty evidence) and lower recurrence (RR 0.28, 95% CI 0.15–0.53; moderate-certainty evidence), but no clear differences in mortality (RR 0.36, 95% CI 0.05–2.43) or overall complications (RR 1.04, 95% CI 0.45–2.39), both with low-certainty evidence. Compared with bronchoscopic balloon tamponade, BAE showed no clear difference in clinical efficacy (RR 0.91, 95% CI 0.80–1.03; low-certainty evidence), recurrence (RR 2.14, 95% CI 0.17–27.10; very-low-certainty evidence), or overall complications (RR 1.15, 95% CI 0.54–2.46; low-certainty evidence).

**Conclusions:**

The comparative effects of BAE varied by treatment strategy. Evidence was more consistent versus pharmacotherapy, while estimates for bronchoscopic balloon tamponade and add-on usual-care designs were less certain. Findings should therefore be interpreted by specific treatment contrast rather than as a single overall effect. Further well-designed multicenter RCTs are needed.

**Systematic Review Registration:**

https://www.crd.york.ac.uk/PROSPERO identifier CRD420250643812.

## 1 Introduction

Hemoptysis is defined as the expectoration of blood originating from the lower respiratory tract, specifically below the level of the glottis (1, 2). Epidemiological studies have identified the most common causes of hemoptysis as respiratory infections, bronchial carcinoma or metastases, and bronchiectasis or cystic fibrosis (3). Massive hemoptysis, however, can quickly and unpredictably lead to death due to airway obstruction and hypoxemia secondary to asphyxiation (4, 5). Current treatment options for hemoptysis include pharmacotherapy, surgery, and endovascular treatment, with bronchial artery embolization (BAE) being an important minimally invasive approach.

Compared to pharmacotherapy and surgery, BAE provides targeted control of bleeding with less tissue injury and shorter recovery time. BAE effectively controls bleeding in 80% to 90% of cases and confers a favorable risk-benefit profile (6). The recurrence rate of hemoptysis after BAE has been reported to range from 10% to 57%, with major complications, such as spinal cord ischemia and cerebral infarction, occurring in 0% to 4.6% of cases (7, 8). Although BAE appears to offer advantages over other treatment modalities, high-quality evidence from randomized controlled trials (RCTs) remains limited.

Several systematic reviews and meta-analyses have evaluated the efficacy and safety of BAE for hemoptysis, but only one included three RCTs, while the others were based exclusively on cohort studies (9–13). Therefore, we conducted a systematic review and meta-analysis of RCTs to evaluate the efficacy and safety of BAE compared with alternative therapeutic modalities. The certainty of evidence was further assessed using the Grading of Recommendations Assessment, Development and Evaluation (GRADE) approach to provide a more robust evaluation of the clinical evidence (14).

## 2 Methods

This systematic review was conducted in accordance with the Preferred Reporting Items for Systematic Reviews and Meta-Analyses (PRISMA) statement (15) and was prospectively registered in the international systematic review registry PROSPERO (https://www.crd.york.ac.uk/PROSPERO) under the registration number CRD420250643812.

### 2.1 Data sources

We performed comprehensive electronic literature searches up to December 29, 2024, without language restrictions, across the following databases: PubMed, Embase, Cochrane Library, and Chinese databases such as China National Knowledge Infrastructure (CNKI), Wanfang Database, China Science and Technology Journal Database (CQVIP), and Sinomed. The primary search terms included “hemoptysis”, “interventional therapy”, “Bronchial artery embolization,” and “BAE.” We additionally hand-searched the reference lists of included studies and relevant prior systematic reviews (9–13) to identify additional potentially eligible trials. An updated search was conducted on June 24, 2026, and no additional eligible RCTs were identified. The search strategy for each database is presented in the Supplementary Materials search strategy.

### 2.2 Eligibility criteria and study selection

We included RCTs that compared the clinical efficacy and safety of BAE with alternative therapeutic modalities for hemoptysis. The comparator strategies included pharmacotherapy, bronchoscopic balloon tamponade, surgery, and usual care in add-on treatment designs. Our primary outcomes were clinical efficacy, hemoptysis recurrence, mortality and overall complications. Hemoptysis recurrence was defined as the recurrence of hemoptysis following the completion of treatment. In accordance with relevant clinical guidelines, we stratified the analysis by follow-up time subgroups as follows: 1 month, 3 months, 6 months, and 12 months (16). The definition of clinical efficacy was not standardized across the studies we included. We thus adopted the study-specific definitions of clinical efficacy provided by the authors in each study for the present analysis. Our secondary outcomes included pulmonary function (forced expiratory volume in one second/forced vital capacity, FEV1/FVC) and arterial blood gas parameters (e.g., partial pressure of arterial oxygen [PaO_2_], partial pressure of arterial carbon dioxide [PaCO_2_], and arterial pH). We excluded ongoing trials and studies for which the full texts were unavailable.

Two reviewers independently conducted title and abstract screening followed by full-text eligibility assessment; conflicts were settled by consensus, with a third reviewer consulted to arbitrate as needed.

### 2.3 Data collection

Two reviewers independently extracted data from all included studies using a pre-designed standardized Excel form. The extracted data were then cross-checked between the two reviewers, and any discrepancies were resolved through discussion and consensus, with a third reviewer consulted when necessary. We extracted data pertaining to key study characteristics, including author names, year of publication, and the country/region of study implementation. Collected patient-related characteristics included age, gender, underlying etiology of hemoptysis, hemoptysis severity, baseline sample size, and the type of embolic materials used in BAE interventions. Additionally, comprehensive details of the experimental BAE intervention and control therapeutic measures were systematically recorded. The core outcome indicators included clinical efficacy, hemoptysis recurrence, overall complications, and all reported adverse events.

### 2.4 Risk of bias assessment

Two reviewers independently evaluated the methodological quality of all included RCTs using the revised Cochrane Risk of Bias tool for randomized trials (RoB 2) (17). This tool encompasses five core bias assessment domains, namely: (1) risk of bias arising from the randomization process; (2) risk of bias due to deviations from the intended interventions; (3) risk of bias due to missing outcome data; (4) risk of bias in measurement of the outcome; (5) risk of bias in selection of the reported result.

### 2.5 Statistical analysis

Meta-analysis was performed using Stata 18 software. We performed random-effects models for all meta-analyses, and data were analyzed using the DerSimonian-Laird method. For binary outcomes (e. g., clinical efficacy, recurrence, mortality and complications), results were presented as relative risk (RR) with 95% confidence intervals (CIs), while continuous outcomes (e. g., time to hemostasis, FEV1/FVC, PaO_2_, PaCO_2_ and PH) were reported as the weighted mean difference (WMD) and 95% CI.

### 2.6 Subgroup analyses and sensitivity analyses

We used Cochran's chi-squared test and the I2 statistic to quantify statistical heterogeneity. The heterogeneity classification criteria recommended by guidelines for meta-analyses of randomized trials (18) were as follows: 1) 0% to 40%: heterogeneity may be negligible; 2) 30% to 60%: may represent moderate heterogeneity; 3) 50% to 90%: may represent substantial heterogeneity; 4) 75% to 100%: considerable heterogeneity. Subgroup analyses were performed according to follow-up time. In accordance with the relevant guidelines, follow-up time was categorized as 1 month, 3 months, 6 months, and 12 months (16). For treatment method, because the comparator interventions represented clinically distinct treatment strategies, comparator-specific analyses were designated as the primary analyses for the main outcomes. These analyses were performed according to treatment contrast, including BAE versus pharmacotherapy, BAE versus bronchoscopic balloon tamponade, BAE versus surgery, and BAE plus usual care versus usual care, where data were available. Differences in treatment effects across comparator types were assessed using tests for between-group differences. Overall analyses combining different comparator strategies were retained only as exploratory secondary syntheses and were not interpreted as estimates of a uniform treatment effect applicable to individual clinical comparisons. We also conducted a sensitivity analysis using the Hartung-Knapp-Sidik-Jonkman method to evaluate its impact on the pooled effect estimates (19).

### 2.7 Publication bias

When at least 10 studies were available for meta-analysis, we assessed publication bias by examining funnel plot asymmetry and conducting Harbord's test (20) for all included binary outcomes.

### 2.8 Certainty of evidence

We used the GRADE approach to evaluate the certainty of evidence separately for each main outcome–treatment contrast, classifying the certainty as high, moderate, low, or very low (14). For the risk-of-bias domain, we considered the methodological limitations of the contributing trials and the relative contribution of studies at high risk of bias to each pooled estimate, with high-risk-of-bias evidence considered potentially dominant when it contributed ≥55% or ≥65% of the meta-analytic weight. Where appropriate, sensitivity analyses excluding high-risk-of-bias studies were performed, and the direction and magnitude of effect estimates before and after exclusion were compared to inform the final risk-of-bias judgment (21). We also calculated 95% prediction intervals to assist in the assessment of inconsistency (22), and assessed imprecision using the optimal information size (OIS) and 95% CI (23).

## 3 Results

### 3.1 Study identification

Our search retrieved 8,000 citations, of which 4,099 citations remained following duplicate removal. After screening these 4,099 titles and abstracts as well as 90 full texts, 20 RCTs were deemed eligible for inclusion in this systematic review (Figure 1).

**Figure 1:**
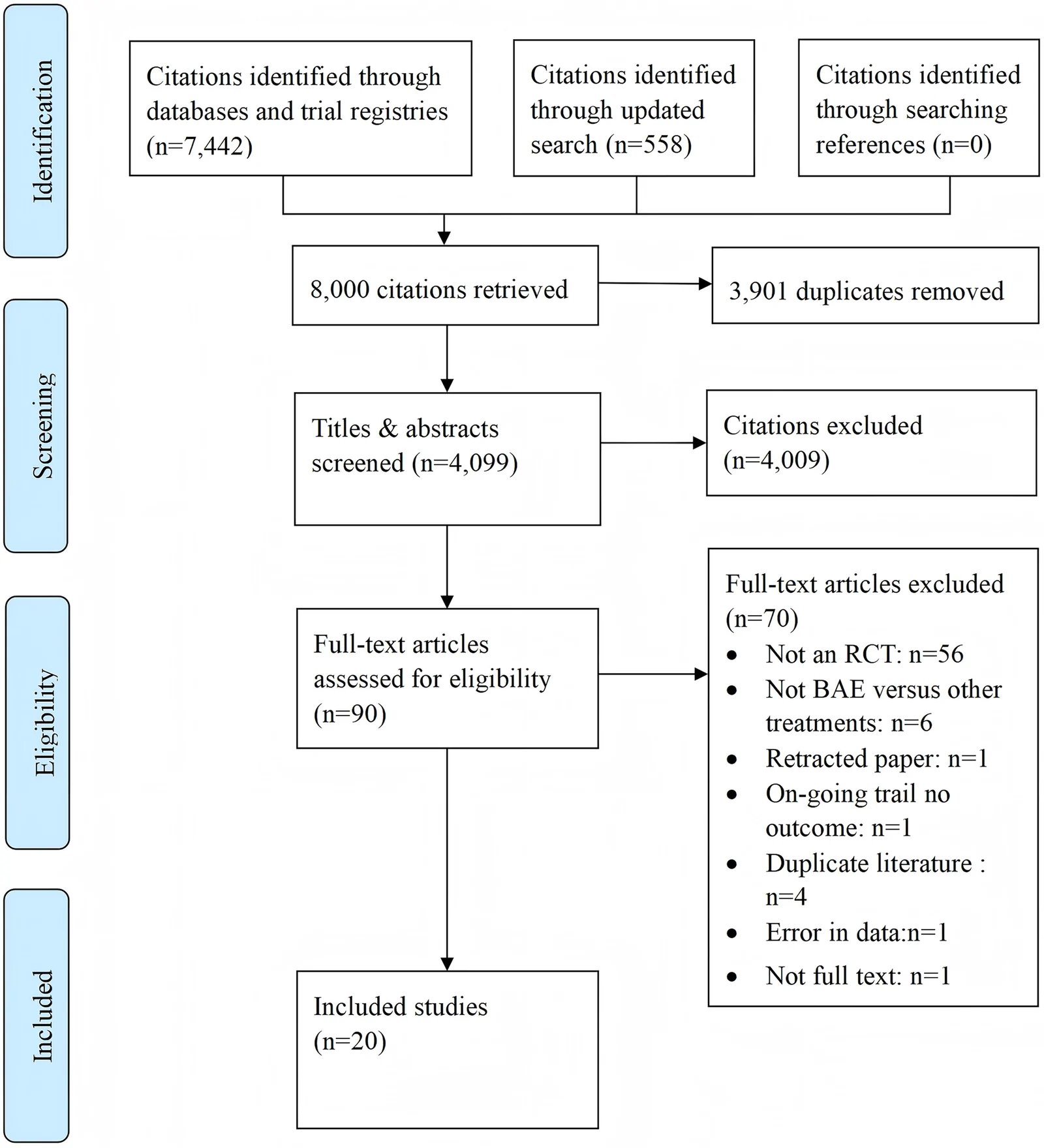
PRISMA flow diagram of study selection.

### 3.2 Characteristics of included studies

From the initially retrieved 8,000 citations, 20 trials with a total of 1,594 patients proved eligible for review, including 1 English-language trial (6) and 19 Chinese-language trials (24–42). The etiology of hemoptysis in these studies included tuberculosis (5 studies), bronchiectasis (3 studies), lung cancer (1 study), cystic fibrosis, pneumonia, and mixed etiologies (10 studies). With respect to hemoptysis severity, 16 involved massive hemoptysis, 1 involved mild hemoptysis, and 3 involved mixed hemoptysis. Additionally, 13 studies compared BAE with pharmacotherapy, 2 compared BAE with bronchoscopic balloon tamponade, 1 compared BAE with surgery, and 4 compared BAE plus usual care versus usual care alone. These key characteristics are summarized in Table 1. Peer-review procedures could be independently verified for the journals publishing 10 of the 20 included studies, including 9 Chinese-language studies and the single English-language study. For the remaining studies, peer-review status was reported by the publication sources but could not be independently verified from specific publicly available documentation (Supplementary eTable 8).

**Table 1:** Baseline characteristics of 20 eligible trials.

Study	Nation	Number of Patients		Age	Female	Severity of hemoptysis	Embolic materials	Etiology of hemoptysis	Follow-up time
		BAE	Other treatment						
Fartoukh 2021 (6)	France	34	38	52.80 ± 6.56	21	Mild Hemoptysis	NR	Bronchiectasis Tuberculosis Lung cancer Pneumonia lung abscess Cryptogenic Tuberculosis sequels	90d
Zhang XB 2022 (28)	China	39	39	56.30 ± 9.39	29	NR	PVA	Bronchiectasis Tuberculosis	60d
Ding XW 2019 (34)	China	30	30	55.20 ± 6.46	25	Massive Hemoptysis	Gelatin sponge	Tuberculosis	NR
Yuan GD 2020 (36)	China	58	59	44.40 ± 6.50	44	Massive Hemoptysis	NR	Tuberculosis	90d
Cai ZY 2020 (35)	China	30	30	51.90 ± 7.31	21	NR	Gelatin sponge	Bronchiectasis	4d
Liu EQ 2013 (24)	China	30	30	23–64	37	Massive Hemoptysis	Spring coil	Bronchiectasis Tuberculosis Lung cancer Vascular malformation	365d
Liu Y 2015 (41)	China	42	42	38.16 ± 14.62	20	Massive Hemoptysis	NR	Tuberculosis	180d
Gao DD 2023 (32)	China	40	40	47.20 ± 8.41	35	Massive Hemoptysis	PVA Gelatin sponge Spring coil	Lung cancer	180d
Tian YH 2014 (33)	China	44	44	51.20 ± 3.20	29	Massive Hemoptysis	PVA Gelatin sponge	Bronchiectasis	10d
Zhang L 2023 (31)	China	30	30	65.80 ± 6.41	25	Massive Hemoptysis	Gelatin sponge Spring coil	Bronchiectasis	3d
Chen HS 2022 (25)	China	30	30	46.80 ± 5.06	16	Massive Hemoptysis	PVA Gelatin sponge	Bronchiectasis	365d
Guan HX 2021 (30)	China	30	30	49.80 ± 10.16	23	Massive Hemoptysis	PVA Gelatin sponge	Bronchiectasis Tuberculosis Lung cancer Cryptogenic	7d
Huang CC 2023 (27)	China	52	52	51.70 ± 17.42	63	Massive Hemoptysis	Gelatin sponge	Bronchiectasis	14d
Zeng LH 2022 (26)	China	41	41	60.40 ± 5.32	49	NR	PVA	Bronchiectasis Tuberculosis Lung cancer	90d
Li HX 2021 (38)	China	30	30	51.40 ± 5.54	19	Massive Hemoptysis	PVA Gelatin sponge	Bronchiectasis Tuberculosis Lung cancer	1d
Li CY 2017 (40)	China	47	47	43.60 ± 5.81	20	Massive Hemoptysis	PVA Gelatin sponge	Tuberculosis	180d
Zou C 2024 (39)	China	90	137	63.60 ± 15.23	77	Massive Hemoptysis	PVA Gelatin sponge	NR	7d
Lin DX 2002 (37)	China	20	22	54.70 ± 13.82	11	Massive Hemoptysis	Gelatin sponge	Bronchiectasis Tuberculosis Lung cancer	180d
Zhang QY 2012 (29)	China	30	30	53.30 ± 15.03	21	Massive Hemoptysis	PVA Gelatin sponge Spring coil	Bronchiectasis Tuberculosis Lung cancer	240d
Xu YH 2019 (42)	China	23	23	43.60 ± 5.77	11	Massive Hemoptysis	PVA	Tuberculosis	180d

### 3.3 Risk of bias

Of the 20 included studies, 12 studies (60%) reported the method of random sequence generation, 1 (5%) implemented allocation concealment, and all 20 (100%) ensured blinding of data collectors and outcome assessors. Blinding of surgeons and patients was not feasible. However, we judged that the lack of blinding was unlikely to exert a substantial impact on the assessed outcomes. The risk of bias attributable to incomplete outcome data and selective reporting was assessed as low across all studies, whereas funding sources were only reported in five studies and remained unclear in the remaining studies. The results from the quality assessment using the Risk of Bias (RoB 2) are summarized in (Supplementary Material eTable 1).

### 3.4 Clinical efficacy

Clinical efficacy was reported in 17 RCTs (24–33, 35, 37–42) involving 1,345 patients. In the primary comparator-specific analyses, moderate-certainty evidence showed higher clinical efficacy with BAE than with pharmacotherapy (RR 1.24, 95% CI 1.18–1.31). Very-low-certainty evidence supported higher clinical efficacy with BAE plus usual care versus usual care (RR 1.27, 95% CI 1.12–1.43), whereas low-certainty evidence showed no clear difference between BAE and bronchoscopic balloon tamponade (RR 0.91, 95% CI 0.80–1.03). Effect estimates differed across comparator types (P for between-group differences <0.001; Figure 2; Table 2).

**Figure 2:**
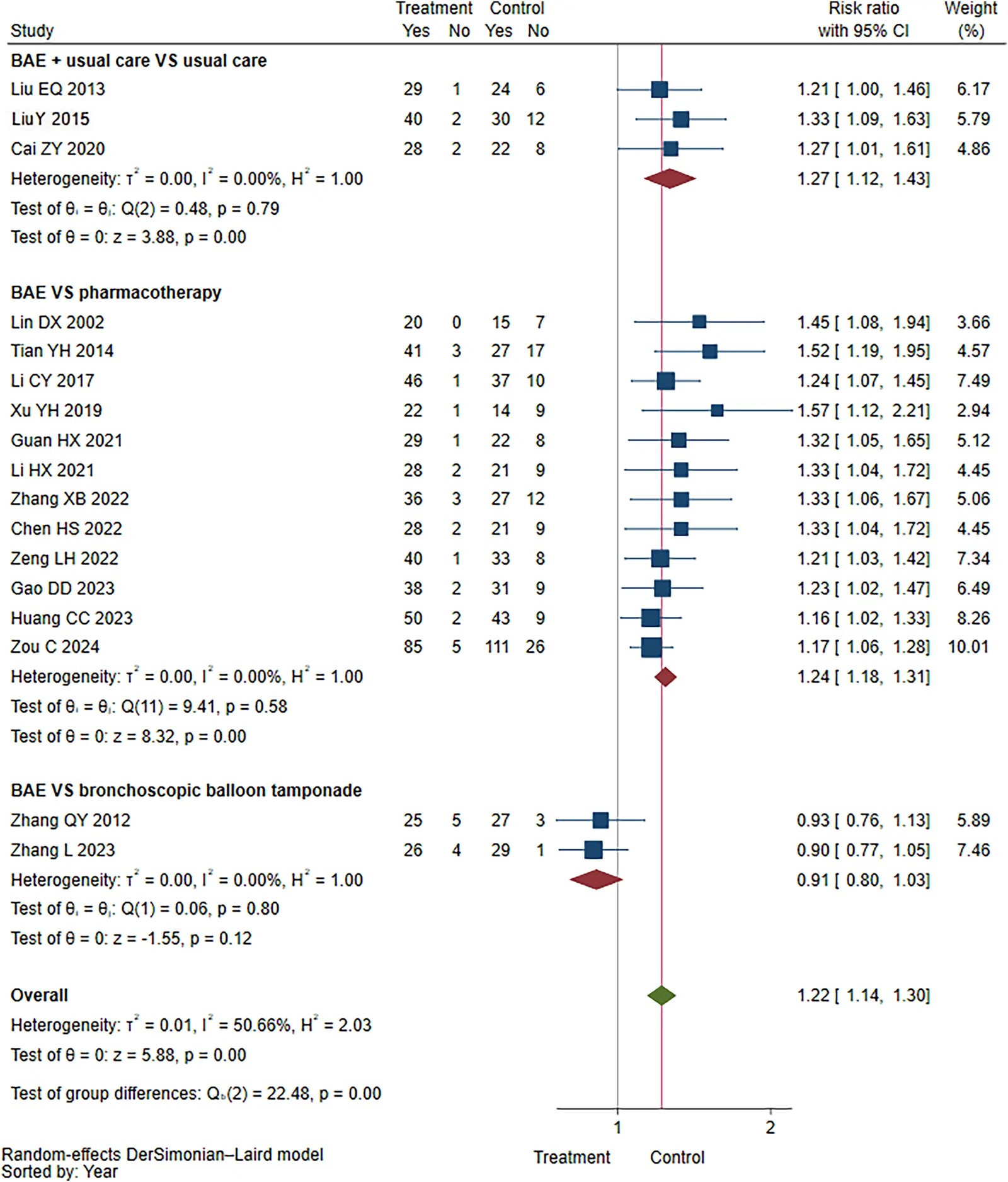
Forest plot of comparator-specific analysis of clinical efficacy.

**Table 2:** GRADE evidence profile of clinical efficacy, recurrence, mortality, and overall complications.

Outcome (No. of trials/ patients)	Treatment contrast	No. of trials	Risk of bias	Inconsistency	Indirectness	Imprecision	Publication bias	Treatment association (95% CI)	Overall certainty of evidence
Clinical efficacy 17 (1,345)	Pharmacotherapy usual care balloon tamponade	12	No*(55.22%)	I^2^=0%	Yes↓	No	No(*p*=0.20)	RR 1.24 (1.18 to 1.31)	Moderate
		3	Yes(74.25%)↓	I^2^=0%	Yes↓	Yes↓	NA	RR 1.27 (1.12 to 1.43)	Very Low
		2	No*(37.71%)	I^2^=0%	Yes↓	Yes↓	NA	RR 0.91 (0.80 to 1.03)	Low
Recurrence 9 (616)	Pharmacotherapy usual care balloon tamponade	4	No*(0%)	I^2^=0%	No	Yes↓	NA	RR 0.26 (0.13 to 0.54)	Moderate
		3	No*(45.65%)	I^2^=0%	No	Yes↓	NA	RR 0.28 (0.15 to 0.53)	Moderate
		2	Yes(55.13%)↓	I^2^=79.41%↓	No	Yes↓	NA	RR 2.14 (0.17 to 27.10)	Very Low
Mortality 5 (356)	Pharmacotherapy usual care	2	No*(0%)	I^2^=0%	No	Yes↓	NA	RR 0.20 (0.05 to 0.90)	Moderate
		Yes(76.15%)↓	I^2^=0%	No	Yes↓	NA	RR 0.36 (0.05 to 2.43)	Low	
Overall complications 13 (924)	Pharmacotherapy usual care balloon tamponade	8	No*(40.54%)	I^2^=0%	No	Yes↓	NA	RR 0.41 (0.26 to 0.63)	Moderate
		3	Yes(60.29%)↓	I^2^=46.17%	No	Yes↓	NA	RR 1.04 (0.45 to 2.39)	Low
		2	Yes(66.98%)↓	I^2^=7.15%	No	Yes↓	NA	RR 1.15 (0.54 to 2.46)	Low

BAE, bronchial artery embolization; RR, elative risk; CI: 95% confidence interval; NA, not applicable due to less than 10 studies; pharmacotherapy: BAE vs. pharmacotherapy; usual care: BAE + usual care vs. usual care; balloon tamponade: BAE vs. bronchoscopic balloon tamponade; ↓:downgrade by one level; *: Not serious, Risk-of-bias judgments incorporated the methodological limitations of the contributing trials, the proportion of meta-analytic weight contributed by high-risk trials, and sensitivity analyses excluding high-risk trials.

In an exploratory secondary analysis pooling these clinically heterogeneous comparator strategies, BAE was associated with higher clinical efficacy (RR 1.22, 95% CI 1.14–1.30; Supplementary Materials eFigure 1). The effect on time to hemostasis was supported by only very low certainty evidence (5 RCTs (27–29, 31, 35) and 362 patients; WMD −0.54, 95% CI −1.84–0.76; Supplementary Materials eFigure 2, eTable 5).

### 3.5 Recurrence

Hemoptysis recurrence was reported in 9 RCTs involving 616 patients (6, 24, 25, 29, 31, 32, 40–42). In the primary comparator-specific analyses, the RR was 0.26 (95% CI 0.13–0.54) for BAE versus pharmacotherapy, 0.28 (95% CI 0.15–0.53) for BAE plus usual care versus usual care, and 2.14 (95% CI 0.17–27.10) for BAE versus bronchoscopic balloon tamponade. No significant difference in effect estimates was detected across comparator types (*P* = 0.29; Figure 3). In an exploratory secondary analysis pooling the different comparator strategies, the overall RR was 0.38 (95% CI 0.21–0.69; Supplementary Materials eFigure 3).

**Figure 3:**
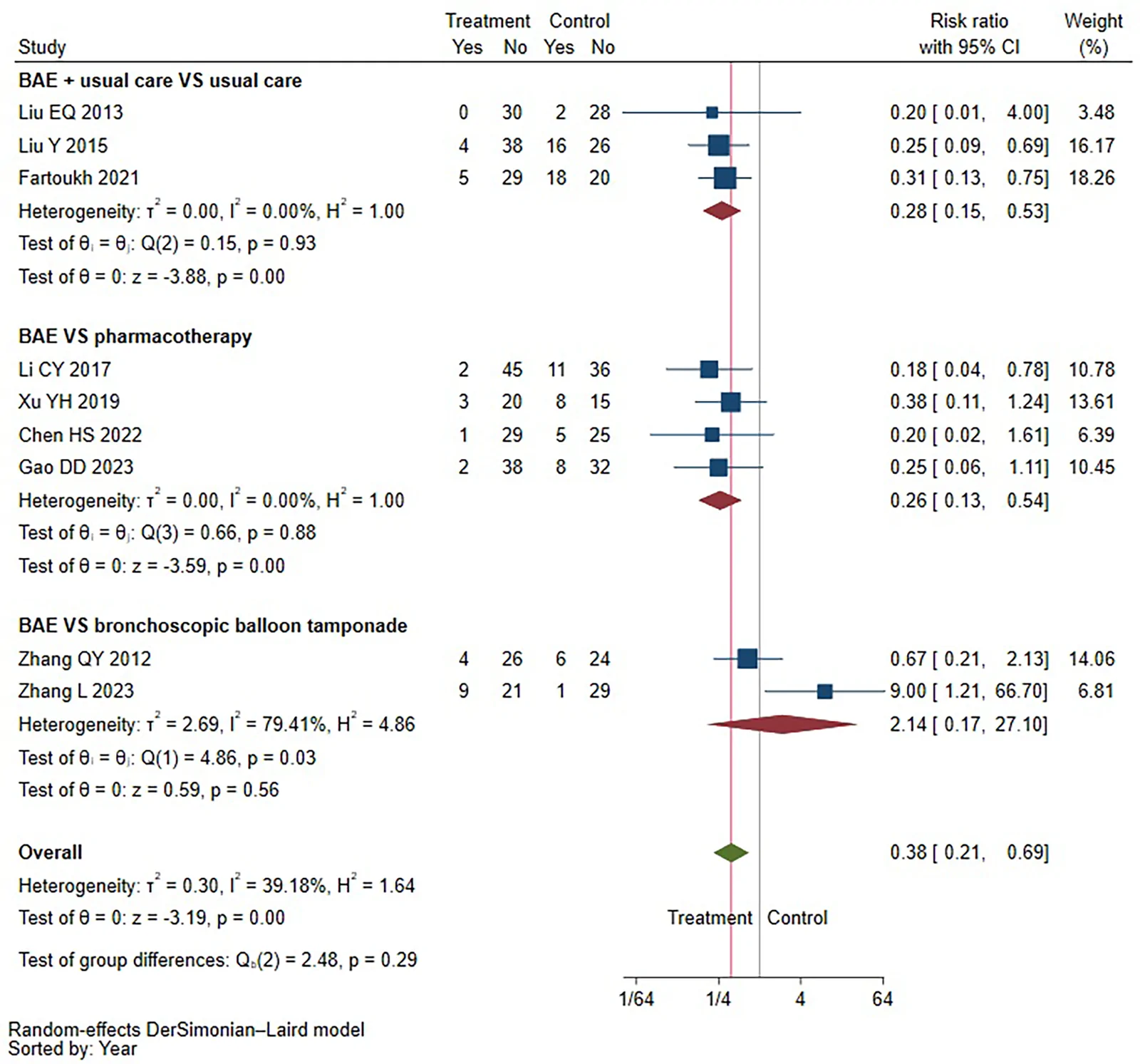
Forest plot of comparator-specific analyses for recurrence.

### 3.6 Overall complications and adverse events

Overall complications were reported in 13 RCTs (6, 24, 26–31, 33–35, 40, 42) involving 924 patients. In the primary comparator-specific analyses, moderate-certainty evidence showed a lower risk of overall complications with BAE versus pharmacotherapy (RR 0.41, 95% CI 0.26–0.63), whereas low-certainty evidence showed no clear difference for BAE plus usual care versus usual care (RR 1.04, 95% CI 0.45–2.39) or for BAE versus bronchoscopic balloon tamponade (RR 1.15, 95% CI 0.54–2.46). Effect estimates differed across comparator types (P for between-group differences =0.02; Figure 4; Table 2). In an exploratory secondary analysis pooling the different comparator strategies, the overall RR was 0.63 (95% CI 0.44–0.91; Supplementary Materials eFigure 4).

**Figure 4:**
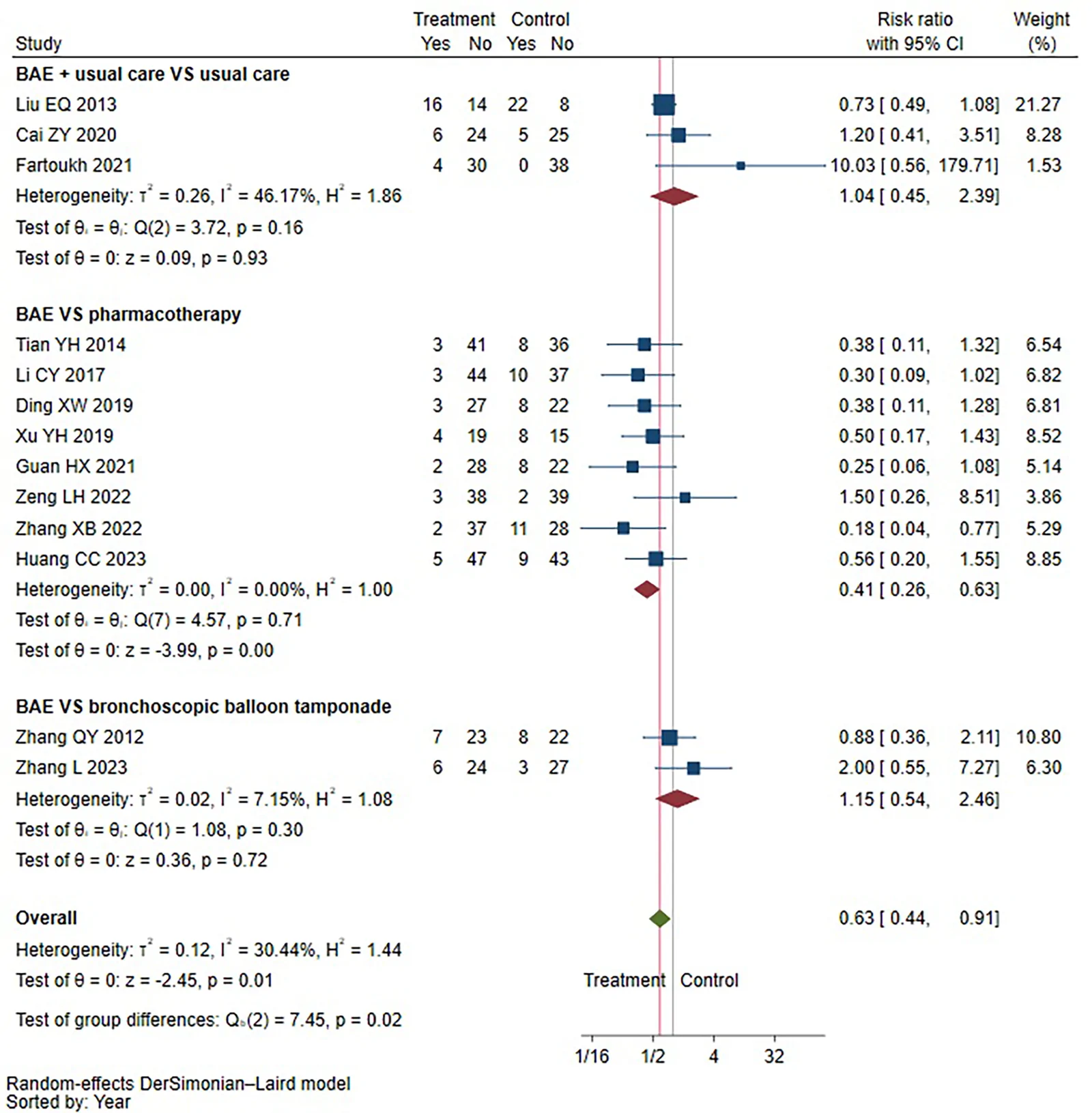
Forest plot of comparator-specific analyses for overall complications.

Mortality was reported in 5 RCTs (6, 24, 40–42) involving 356 patients. In the primary comparator-specific analyses, moderate-certainty evidence showed lower mortality with BAE versus pharmacotherapy (RR 0.20, 95% CI 0.05–0.90), whereas low-certainty evidence showed no clear difference for BAE plus usual care versus usual care (RR 0.36, 95% CI 0.05–2.43). No significant difference in effect estimates was detected between treatment contrasts (*P* = 0.64; Figure 5; Table 2). In an exploratory secondary analysis pooling the different comparator strategies, the overall RR was 0.25 (95% CI 0.08–0.81; Supplementary materials eFigure 5).

**Figure 5:**
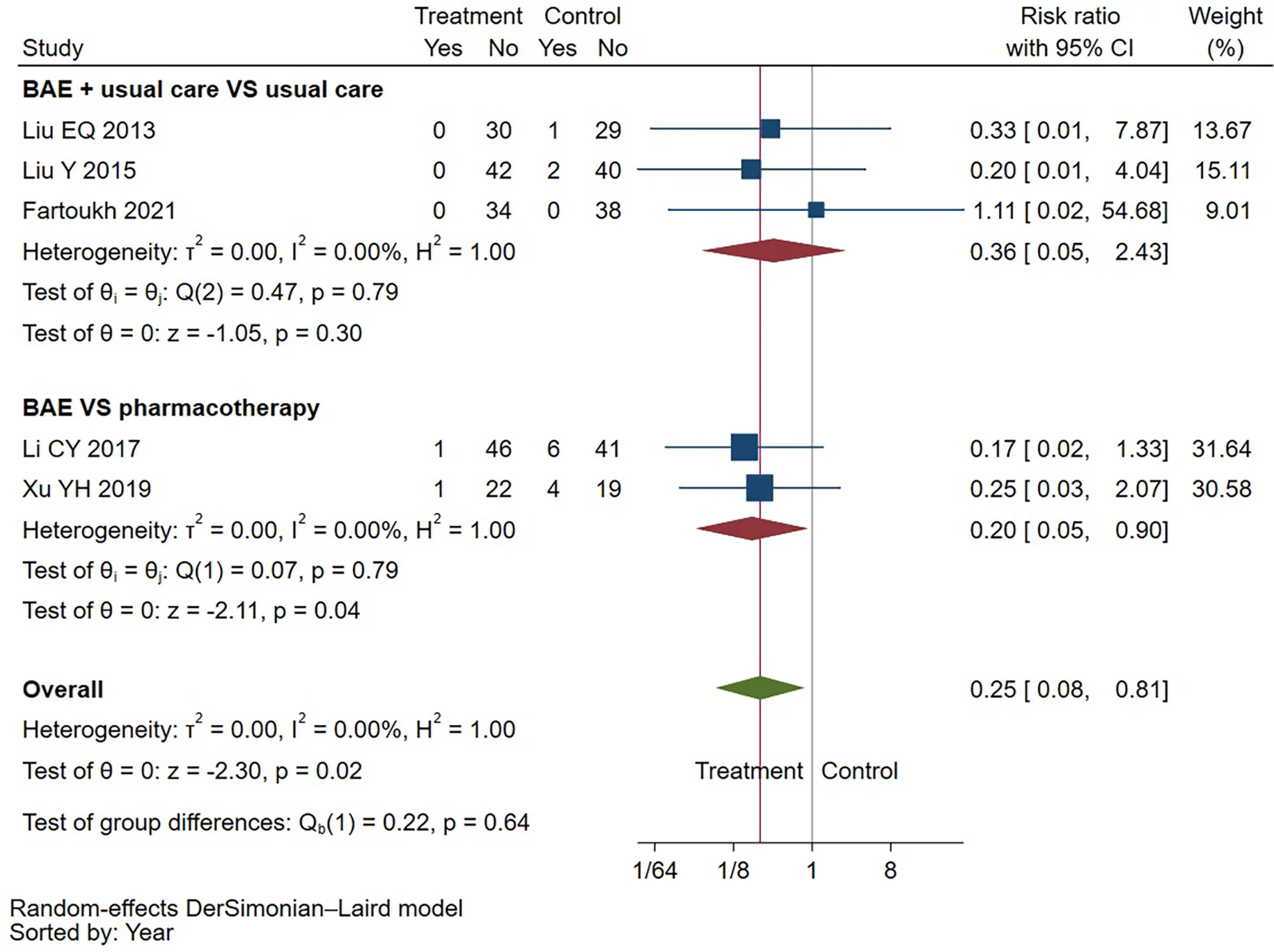
Forest plot of comparator-specific analyses for mortality.

Moderate-certainty evidence also suggested lower risks of nausea and vomiting (5 RCTs (30, 33, 34, 40, 42), 348 patients; RR 0.11, 95% CI 0.03–0.40; Supplementary Materials eFigure 6) and abdominal pain (3 RCTs (34, 40, 42), 200 patients; RR 0.28, 95% CI 0.05–1.67). The effect of BAE on chest pain (9 RCTs (26–31, 35, 40, 42) and 641 patients; RR 0.54, 95% CI 0.23–1.23; Supplementary Materials eFigure 7), and palpitations (4 RCTs (27, 35, 40, 42) and 304 patients; RR 0.62, 95% CI 0.18–2.15) were supported by moderate certainty evidence. The effect on cough (3 RCTs (29, 40, 42) and 200 patients; RR 0.90, 95% CI 0.05–15.51) was supported by low certainty evidence. However, the moderate evidence of fever (7 RCTs (27–31, 34, 35) and 482 patients; RR 1.28, 95% CI 0.48–3.40; Supplementary Materials eFigure 8) and the low evidence of chest distress (2 RCTs (26, 33) and 170 patients; RR 1.23, 95% CI 0.34–4.48) indicated higher incidence of these adverse reactions in BAE treatment compared with the control group (Supplementary eTable 4).

### 3.7 Additional outcomes

Low certainty evidence from 5 RCTs (28, 32, 36, 40, 42) encompassing 415 patients showed that post-treatment pulmonary function in the BAE group was superior to that in the control group (FEV1/FVC, WMD 7.62, 95% CI 4.80–10.45; Supplementary Materials eFigure 9). The effect of BAE on arterial blood gas parameters including PaO2 (4 RCTs (32, 36, 40, 42) and 337 patients; WMD 1.38, 95% CI 0.48–2.28), PaCO2 (4 RCTs (32, 36, 40, 42) and 337 patients; WMD −1.00, 95% CI −1.68 to −0.32), and pH (4 RCTs (32, 36, 40, 42) and 337 patients; WMD 0.11, 95% CI −0.06–0.29), was supported by only low certainty evidence (Supplementary eTable 5).

### 3.8 Subgroup analyses and sensitivity analyses

Subgroup analyses according to follow-up time are presented in the Supplementary Materials. Comparator-specific analyses of the major outcomes showed differences in effect estimates across comparator types for clinical efficacy and overall complications, but not for recurrence or mortality (Supplementary Materials eTables 2–S3; eFigure 1; eFigures 3–S5). Sensitivity analyses using the Hartung–Knapp–Sidik–Jonkman method yielded effect estimates of similar direction and magnitude to those obtained with the DerSimonian–Laird method. However, for some outcomes (e.g., overall complications, PaO₂, and PaCO₂), the confidence intervals became wider and crossed the null value, indicating reduced statistical certainty (Supplementary Materials eTables 6 to 7). Sensitivity analyses excluding trials at high risk of bias were additionally performed for the main outcomes. For several treatment contrasts, the effect estimates were similar after exclusion of high-risk trials, whereas for others the estimates changed materially or only one lower-risk trial remained. These analyses informed the GRADE risk-of-bias judgments and are presented in Supplementary eFigures 10–S13 and eTable 9. Because the exploratory overall analyses combined clinically distinct comparator strategies, these pooled estimates should not be interpreted as uniform treatment effects or used to guide decisions for specific clinical comparisons.

## 4 Discussion

### 4.1 Principal findings

To our knowledge, this is the first systematic review and meta-analysis to synthesize evidence exclusively from randomized controlled trials evaluating bronchial artery embolization (BAE) for hemoptysis. By minimizing the influence of confounding inherent in observational studies and evaluating evidence certainty using the GRADE approach, this review provides a more rigorous assessment of the comparative efficacy and safety of BAE.

In this meta-analysis of 20 RCTs involving 1,594 patients, the primary comparator-specific analyses showed that the comparative effects of BAE varied according to the treatment contrast. Compared with pharmacotherapy, BAE was associated with higher clinical efficacy, lower recurrence and mortality, and fewer overall complications. BAE plus usual care was associated with higher clinical efficacy and lower recurrence than usual care alone, whereas no clear differences were observed for mortality or overall complications. Comparisons between BAE and bronchoscopic balloon tamponade did not show clear differences for several major outcomes, although the available evidence was limited. The certainty of evidence varied across outcomes and treatment contrasts. For clinical efficacy, variation in the definitions and assessment criteria across trials introduced clinical heterogeneity and indirectness in outcome measurement, warranting a one-level downgrade in the certainty of evidence.

The between-comparator differences observed for clinical efficacy and overall complications further support interpretation within specific treatment contrasts rather than reliance on a single overall pooled estimate. In contrast, no significant between-comparator differences were detected for recurrence or mortality, although the number of studies was limited for several treatment contrasts. Overall pooled analyses across the different comparator strategies were considered exploratory and should not be interpreted as uniform treatment effects applicable to specific clinical comparisons. In the exploratory overall analysis, fewer overall complications were observed with BAE; however, sensitivity analysis using the Hartung–Knapp–Sidik–Jonkman method produced wider confidence intervals, and statistical significance was not retained. This finding should therefore be interpreted cautiously.

The favorable effects observed in several comparator-specific analyses are biologically plausible. Clinically significant hemoptysis usually originates from hypertrophied bronchial arteries and abnormal systemic collateral vessels resulting from chronic pulmonary inflammation (43, 44). By selectively embolizing these pathological vessels, BAE directly eliminates the bleeding source and interrupts persistent collateral circulation, thereby improving immediate hemostasis and reducing the risk of recurrent bleeding. Advances in endovascular techniques, including superselective microcatheterization, more comprehensive embolization of collateral vessels, and the increasing use of permanent embolic materials, may have further contributed to improved long-term outcomes.

No significant difference was observed in time to hemostasis. This finding was supported by very-low-certainty evidence based on a limited number of studies. Variations in the definition and assessment of hemostasis across studies may have further increased uncertainty. Because most included trials enrolled patients with moderate-to-severe or massive hemoptysis, the applicability of these findings to patients with mild or intermittent bleeding remains uncertain.

### 4.2 Relation to previous studies

To date, five English-language systematic reviews related to BAE for hemoptysis have been published (9–13). The most recent review, published in 2024, included three RCTs together with nine cohort studies (11), whereas the remaining reviews were based exclusively on observational evidence. In contrast, the present review synthesized evidence solely from randomized controlled trials and incorporated 19 additional RCTs that had not been evaluated previously. This discrepancy is likely attributable primarily to differences in database coverage, as our search included four major Chinese databases (CNKI, Wanfang, CQVIP, and SinoMed) in addition to international databases. Nevertheless, the predominance of Chinese-language RCTs and the methodological reporting limitations of some of these studies should be considered when interpreting the findings.

Our comparator-specific findings are broadly consistent with previous evidence suggesting beneficial effects of BAE for hemoptysis. Compared with earlier reports, recurrence following BAE appears to have declined over time, likely reflecting advances in interventional practice, including routine preprocedural CT angiography, superselective microcatheterization, more complete embolization of non-bronchial systemic collateral vessels, and wider adoption of permanent embolic agents. Concurrent improvements in disease-specific medical therapy, such as standardized anti-tuberculosis and antifungal treatment, may also contribute to improved long-term outcomes.

The effectiveness of BAE should also be interpreted in the context of the underlying etiology of hemoptysis. Patients included in the present review had diverse causes of bleeding, including bronchiectasis, tuberculosis, malignancy, and other pulmonary diseases (45). Because patients with different etiologies of hemoptysis exhibit distinct patterns of vascular remodeling and collateral vessel formation, the magnitude and durability of treatment benefit may vary across disease populations. Future randomized studies should therefore evaluate treatment effects according to underlying disease etiology.

From a clinical perspective, our findings further support current guideline recommendations that BAE should be considered an early therapeutic option for patients with moderate-to-massive hemoptysis, recurrent bleeding, or those unsuitable for surgery. Surgery remains appropriate for selected patients with localized resectable lesions, whereas bronchoscopy continues to play an essential role in airway protection, bleeding localization, and etiological diagnosis. These approaches should therefore be regarded as complementary components of multidisciplinary hemoptysis management rather than competing treatment strategies (46).

The comparator interventions represented distinct therapeutic strategies, including pharmacotherapy, bronchoscopic balloon tamponade, surgery, and usual care in add-on treatment designs. Accordingly, comparator-specific analyses were used as the primary basis for interpretation, whereas overall pooled analyses across these heterogeneous strategies were considered exploratory. Compared with pharmacotherapy, BAE was associated with favorable effects across several major outcomes, whereas evidence from comparisons with bronchoscopic balloon tamponade and surgery was more limited. The add-on comparisons of BAE plus usual care versus usual care provided evidence on the incremental effect of BAE when added to background treatment. Further direct randomized comparisons between BAE and alternative interventional strategies would help define their relative roles in the management of hemoptysis.

### 4.3 Strengths, limitations, and future perspectives

The principal strength of this review is the exclusive inclusion of randomized controlled trials, thereby reducing the influence of selection bias and confounding inherent in observational studies. In addition, methodological quality and evidence certainty were systematically evaluated using the RoB 2 tool, sensitivity analyses, and the GRADE approach.

Several limitations should be acknowledged. First, although only randomized trials were included, some studies had methodological limitations, particularly inadequate reporting of randomization procedures, allocation concealment, and blinding, which may have affected the reliability of several outcome estimates. These methodological limitations, together with the contribution of high-risk-of-bias trials and the results of sensitivity analyses excluding such trials, were considered in the GRADE risk-of-bias judgments. In addition, although peer-review procedures could be independently verified for the journals publishing half of the included studies, such verification was unavailable for the remaining studies, which should be considered when interpreting the evidence base. Second, most included studies were conducted in China, where tuberculosis and post-tuberculosis sequelae remain common causes of hemoptysis. Consequently, the geographical and etiological distribution of the available randomized evidence may not fully reflect that of other regions, particularly settings in which malignancy or other causes account for a greater proportion of hemoptysis. Differences in disease spectrum, healthcare systems, and clinical practice may therefore limit the generalizability of the effect estimates to such populations. Nevertheless, the included trials were not restricted to a single etiology, and the fundamental therapeutic mechanism of BAE—occlusion of the culprit systemic arteries to control bleeding—is not expected to differ substantially according to geographical setting or underlying etiology. Therefore, while caution is warranted when extrapolating the magnitude of the observed effects to populations with substantially different etiological profiles, further geographically and etiologically diverse RCTs are needed to strengthen the external validity of the evidence. These concerns were considered primarily in relation to indirectness and generalizability rather than risk of bias. Third, although exploratory overall analyses were retained for completeness, they combined clinically heterogeneous comparator strategies and therefore should not be interpreted as estimates directly applicable to any single treatment comparison. In addition, the number of trials was limited for some treatment contrasts, particularly comparisons with bronchoscopic balloon tamponade and surgery, resulting in less precise comparator-specific estimates. Fourth, insufficient reporting prevented subgroup analyses according to disease etiology, embolic materials, and embolization techniques, limiting exploration of potential treatment-effect modifiers. Fifth, sensitivity analyses using the Hartung–Knapp–Sidik–Jonkman method yielded wider confidence intervals than the DerSimonian–Laird approach, with the confidence intervals including the null for some secondary outcomes.

The certainty of evidence varied across outcomes and treatment contrasts, with more consistent evidence for comparisons with pharmacotherapy and lower or very-low-certainty evidence for several other comparisons. Future multicenter randomized trials with standardized outcome definitions, longer follow-up, and stratification according to disease etiology are needed to strengthen the evidence base and determine the optimal embolization strategies for different patient populations.

## 5 Conclusions

In conclusion, this systematic review and meta-analysis of RCTs showed that the comparative effects of bronchial artery embolization for hemoptysis varied across treatment strategies. Comparator-specific analyses showed more consistent evidence of benefit when BAE was compared with pharmacotherapy, whereas evidence for add-on usual-care designs and bronchoscopic balloon tamponade was less certain for several outcomes. Overall pooled estimates across heterogeneous comparator strategies should be regarded as exploratory rather than as uniform treatment effects applicable to specific clinical comparisons. Further well-designed multicenter randomized controlled trials with standardized outcome measures and longer follow-up are needed to evaluate the comparative efficacy and safety of BAE across diverse patient populations.

## Data Availability

The original contributions presented in the study are included in the article/Supplementary Material, further inquiries can be directed to the corresponding authors.

## References

[1] Pizarro C Meyer C Schmidt J Skowasch D. [Haemoptysis]. Pneumologie (Stuttgart, Germany). (2023) 77(3):173–83. 10.1055/a-1854-3022 36918017

[2] Dudha M Lehrman S Aronow WS Rosa J. Hemoptysis: diagnosis and treatment. Compr Ther. (2009) 35(3-4):139–49. 20043609

[3] Ittrich H Bockhorn M Klose H Simon M. The diagnosis and treatment of hemoptysis. Dtsch Arztebl Int. (2017) 114(21):371–81. 10.3238/arztebl.2017.0371 28625277 PMC5478790

[4] Prey B Francis A Williams J Krishnadasan B. Evaluation and treatment of massive hemoptysis. Surg Clin North Am. (2022) 102(3):465–81. 10.1016/j.suc.2021.11.002 35671767

[5] Noseworthy TW Anderson BJ. Massive hemoptysis. CMAJ. (1986) 135(10):1097–9. 3533242 PMC1491768

[6] Fartoukh M Demoule A Sanchez O Tuffet S Bergot E Godet C. Randomised trial of first-line bronchial artery embolisation for non-severe haemoptysis of mild abundance. BMJ Open Respir Res. (2021) 8(1):e000949. 10.1136/bmjresp-2021-000949 34088727 PMC8183216

[7] Le Tat T Carlier R Zhang N Bouchet A Amar R Zhou J. Endovascular management of life-threatening hemoptysis in primary lung cancer: a retrospective study. Cardiovasc Interv Radiol. (2023) 46(7):891–900. 10.1007/s00270-023-03488-w 37311841

[8] Pathak V Stavas JM Ford HJ Austin CA Aris RM. Long-term outcomes of the bronchial artery embolization are diagnosis dependent. Lung India. (2016) 33(1):3–8. 10.4103/0970-2113.173059 26933299 PMC4748661

[9] Zheng Z Zhuang Z Yang M Luo J Zhang W Yan Z. Bronchial artery embolization for hemoptysis: a systematic review and meta-analysis. J Interv Med. (2021) 4(4):172–80. 10.1016/j.jimed.2021.08.003 35586385 PMC8947981

[10] Panda A Bhalla AS Goyal A. Bronchial artery embolization in hemoptysis: a systematic review. Diagn Interv Radiol. (2017) 23(4):307–17. 10.5152/dir.2017.16454 28703105 PMC5508955

[11] Fan S Cheng X Wang X Liu Y He W Chen H. Bronchial artery embolization versus conservative treatment for hemoptysis: a systematic review and meta-analysis. BMC Pulm Med. (2024) 24(1):428. 10.1186/s12890-024-03244-x 39215233 PMC11365234

[12] Tao Y Li J Su R Zhou M Zhu H Sun Z. The efficacy, safety, and related factors of bronchial artery embolization for hemoptysis: a systematic review and meta-analysis with subgroup analysis. Cardiovasc Diagn Ther. (2024) 14(5):85977–877. 10.21037/cdt-24-157 PMC11538829 39513143

[13] Karlafti E Tsavdaris D Kotzakioulafi E Kougias L Tagarakis G Kaiafa G. Which is the best way to treat massive hemoptysis? A systematic review and meta-analysis of observational studies. J Pers Med. (2023) 13(12):1649. 10.3390/jpm13121649 38138876 PMC10744930

[14] Guyatt G Agoritsas T Brignardello-Petersen R Mustafa RA Rylance J Foroutan F. Core GRADE 1: overview of the core GRADE approach. Br Med J. (2025) 389:e081903. 10.1136/bmj-2024-081903 40262844

[15] Moher D Liberati A Tetzlaff J Altman DG Group TP. Preferred reporting items for systematic reviews and meta-analyses: the PRISMA statement. PLoS Med. (2009) 6(7):e1000097. 10.1371/journal.pmed.1000097 19621072 PMC2707599

[16] Special Committee on Clinical Guidelines, Chinese College of Interventional Physicians, Chinese Medical Doctor Association. [Expert consensus on bronchial artery embolization for hemoptysis]. Chin J Intern Med. (2025) 64(03):191–9. 10.3760/cma.j.cn112138-20241011-00674

[17] Sterne JAC Savović J Page MJ Elbers RG Blencowe NS Boutron I. Rob 2: a revised tool for assessing risk of bias in randomised trials. Br Med J. (2019) 366:l4898. 10.1136/bmj.l4898 31462531

[18] Higgins JPT Thomas J Chandler J Cumpston M Li T Page MJ. Cochrane Handbook for Systematic Reviews of Interventions. Version 6.4. Cochrane (2023). Available online at: https://www.training.cochrane.org/handbook (Accessed August, 2023).

[19] IntHout J Ioannidis JP Borm GF. The hartung-knapp-sidik-jonkman method for random effects meta-analysis is straightforward and considerably outperforms the standard DerSimonian-laird method. BMC Med Res Methodol. (2014) 14(1):25. 10.1186/1471-2288-14-25 24548571 PMC4015721

[20] Harbord RM Egger M Sterne JAC. A modified test for small-study effects in meta-analyses of controlled trials with binary endpoints. Stat Med. (2006) 25(20):3443–57. 10.1002/sim.2380 16345038

[21] Guyatt G Wang Y Eachempati P Iorio A Murad MH Hultcrantz M. Core GRADE 4: rating certainty of evidence—risk of bias, publication bias, and reasons for rating up certainty. Br Med J. (2025) 389:e083864. 10.1136/bmj-2024-083864 40360206

[22] Guyatt G Schandelmaier S Brignardello-Petersen R De Beer H Prasad M Murad MH. Core GRADE 3: rating certainty of evidence—assessing inconsistency. Br Med J. (2025) 389:e081905. 10.1136/bmj-2024-081905 40328467

[23] Guyatt G Zeng L Brignardello-Petersen R Prasad M De Beer H Murad MH. Core GRADE 2: choosing the target of certainty rating and assessing imprecision. Br Med J. (2025) 389:e081904. 10.1136/bmj-2024-081904 40300802

[24] Liu EQ Wei GY Liao XY Xu MF Luo XP. Clinical study on the treatment of massive hemoptysis with superselective pulmonary artery embolization. Mod Diagn Treat. (2013) 24(10):2329–30. 10.3969/j.issn.1001-8174.2013.10.128

[25] Chen HS Ding C Shuai ZC. Analysis of the difference in the efficacy of conservative treatment and bronchial artery embolization intervention in major hemoptysis. Med Theory Pract. (2022) 35(20):3477–9. 10.19381/j.issn.1001-7585.2022.20.020

[26] Zeng LH Gu JQ Sheng ZL Huang L Gao JH Xiao BS. [Clinical effect of arterial interventional embolization hemostasis in the treatment of middle-aged and elderly patients with hemoptysis]. Chin Med Innov. (2022) 19(16):71–4. 10.3969/j.issn.1674-4985.2022.16.016

[27] Huang CC Gu HY Shi GL. [Effect of interventional embolization on bleeding control rate in patients with bronchiectasis and major hemoptysis]. Chin Foreign Med Care. (2023) 42(5):94–8. 10.16662/j.cnki.1674-0742.2023.05.094

[28] Zhang XB Liu M Song JC. [Clinical study of interventional embolization in the treatment of tuberculosis bronchiectasis and hemoptysis]. Gansu Sci Tech Horizons. (2022) 51(2):91–3. 10.3969/j.issn.1672-6375.2022.02.027

[29] Zhang QY Liu XY Zhang HG Peng YB Luo HH Li ZC. Clinical study on the treatment of massive hemoptysis in the respiratory tract with endobronchial balloon tamponade via bronchoscopy. China Healthc Front. (2012) 7(21):3–4. 10.3969/j.issn.1673-5552.2012.21.0002

[30] Guan HX Huang H Dong ZN Xu PC. Discussion on the effect of using bronchial artery embolization in the treatment of hemoptysis. Contemp Med Theory Ser. (2021) 19(21):76–8. 10.3969/j.issn.2095-7629.2021.21.035

[31] Zhang L Chen H Wang K. Observation of the clinical effect of bronchoscopic intraairway balloon compression and bronchial artery embolization in the treatment of bronchiectasis and hemoptysis. J Aerosp Med. (2023) 34(11):1298–300. 10.3969/j.issn.2095-1434.2023.11.007

[32] Gao DD Jiang HY Zhang HD. The effect of selective bronchial artery embolization in the treatment of lung cancer and its effects on lung function and arterial blood gas. Great Doctor. (2023) 8(1):77–9. 10.3969/j.issn.2096-2665.2023.01.024

[33] Tian YH Ma Y Chen SW. Observation of the efficacy of selective bronchial artery embolization in the treatment of bronchiectasis caused by hemoptysis. Chin Pract Med. (2014) 9(30):72–3. 10.14163/j.cnki.11-5547/r.2014.30.279

[34] Ding XW Huang Y Zhang YL. Efficacy and safety analysis of bronchial artery embolization intervention in the treatment of tuberculosis with diabetic hemoptysis. Electronic Journal Clinical Medical Literature. (2019) 6(50):26. 10.16281/j.cnkijocml.2019.50.017

[35] Cai ZY Ma MY Zhang SB. Clinical study of bronchial artery embolization combined with drug treatment of hemoptysis caused by bronchiectasis. Clin Med Eng. (2020) 27(2):193–4. 10.3969/j.issn.1674-4659.2020.02.0193

[36] Yuan GD Wen JL Li J. Effects of bronchial artery embolization combined with integrated nursing on pulmonary function and arterial blood gas in patients with pulmonary tuberculosis and hemoptysis. Electron J Mod Med Health Res. (2020) 4(21):106–8.

[37] Lin DX Gu JP Gao W. The clinical value of bronchial artery embolization in the rescue of acute macroptysis. Chin J Emerg Med. (2002) (1):50. 10.3760/j.issn:1671-0282.2002.01.018

[38] Li HX Wu DY Yang XY. Clinical application of bronchial artery embolization in patients with major hemoptysis. Jiayou Yunbao. (2021) 3(3):160.

[39] Zou C Ge KY Jiang XD Chen XF Yu C Cheng BL. Observation of the efficacy of bronchial artery embolization in the treatment of major hemoptysis. Chin Pract Med. (2024) 19(10):57–9. 10.14163/j.cnki.11-5547/r.2024.10.013

[40] Li CY Sun LJ Cao W Zhu DW. Clinical efficacy of bronchial artery embolization in the treatment of pulmonary tuberculosis and its impact on lung function. J Guangxi Med Univ. (2017) 34(3):434–8. 10.16190/j.cnki.45-1211/r.2017.03.029

[41] Liu Y Xu Y Chen ZH. Clinical research and analysis of bronchial artery embolization in the treatment of pulmonary tuberculosis hemoptysis. Chin J Microecol. (2015) 27(9):1079–1080 + 1083. 10.13381/j.cnki.cjm.201509025

[42] Xu YH Cai KS Tong LR Wang J. The effect of bronchial artery embolization in the treatment of pulmonary tuberculosis and its effect on lung function in patients. Med Theory Pract. (2019) 32(10):1514–6. 10.19381/j.issn.1001-7585.2019.10.028

[43] Sopko DR Smith TP. Bronchial artery embolization for hemoptysis. Semin Interv Radiol. (2011) 28(1):48–62. 10.1055/s-0031-1273940 PMC3140255 22379276

[44] Chun JY Morgan R Belli AM. Radiological management of hemoptysis: a comprehensive review of diagnostic imaging and bronchial arterial embolization. CardioVasc Interv Radiol. (2010) 33(2):240–50. 10.1007/s00270-009-9788-z 20058006

[45] Larici AR Franchi P Occhipinti M Contegiacomo A Del Ciello A Calandriello L. Diagnosis and management of hemoptysis. Diagn Interv Radiol. (2014) 20(4):299–309. 10.5152/dir.2014.13426 24808437 PMC4463269

[46] Kettenbach J Ittrich H Gaubert JY Gebauer B Vos JA. CIRSE standards of practice on bronchial artery embolisation. Cardiovasc Interv Radiol. (2022) 45(6):721–32. 10.1007/s00270-022-03127-w PMC9117352 35396612

