# Supplementary material for: Efficacy and safety of bronchial artery embolization in the treatment of hemoptysis:a systematic review and meta-analysis of RCTs
Source: Front Med (Lausanne). 2026 Oct 21;13:1959450. doi: 10.3389/fmed.2026.1959450 (PMC13733285; doi:10.3389/fmed.2026.1959450)
Supplement: Supplementary file 1 [file Supplementaryfile1.docx]

**Supplementary Material**

[eTable 1 Risk of bias assessment of 20 eligible randomized clinical trials	2](#_Toc15279)

[eTable 2 Subgroup analyses for clinical efficacy, recurrence, and mortality	6](#_Toc27093)

[eTable 3 Subgroup analyses for overall complications, nausea/vomiting, fever, and chest pain	7](#_Toc16126)

[eTable 4 GRADE evidence profile of adverse events	8](#_Toc14947)

[eTable 5 GRADE evidence profile of time to hemostasis, FEV1/FVC, PaO2, PaCO2, and PH	10](#_Toc19075)

[eTable 6 Sensitivity analysis for binary outcomes	11](#_Toc31331)

[eTable 7 Sensitivity analysis for continuous outcomes	12](#_Toc21620)

[eTable 8 Journal Information and Impact Factors of the 19 Chinese References	13](#_Toc10721)

[eTable 9 Qualitative justification for GRADE risk-of-bias judgments for the main outcomes	15](#_Toc23725)

[eFig. 1 Forest plot of the exploratory overall analysis for clinical efficacy	17](#_Toc20798)

[eFig. 2 Forest plot of the exploratory overall analysis for time to hemostasis	18](#_Toc6687)

[eFig. 3 Forest plot of the exploratory overall analysis for recurrence	19](#_Toc9076)

[eFig. 4 Forest plot of the exploratory overall analysis for overall complications	20](#_Toc5240)

[eFig. 5 Forest plot of the exploratory overall analysis for mortality	21](#_Toc12179)

[eFig. 6 Forest plot of the exploratory overall analysis for Nausea/Vomiting	22](#_Toc5184)

[eFig. 7 Forest plot of the exploratory overall analysis for chest pain	23](#_Toc746)

[eFig. 8 Forest plot of the exploratory overall analysis for fever	24](#_Toc25802)

[eFig. 9 Forest plot of the exploratory overall analysis for FEV1/FVC	25](#_Toc27069)

[eFig. 10 Sensitivity analysis excluding trials at high risk of bias for clinical efficacy	26](#_Toc32415)

[eFig. 11 Sensitivity analysis excluding trials at high risk of bias for recurrence	27](#_Toc10875)

[eFig. 12 Sensitivity analysis excluding trials at high risk of bias for mortality	28](#_Toc9807)

[eFig. 13 Sensitivity analysis excluding trials at high risk of bias for overall complications	29](#_Toc14286)

[eFig.14 Funnel plot for clinical efficacy (BAE vs pharmacotherapy)	30](#_Toc10960)

[eAppendix 1: Search Strategies	31](#_Toc30715)

# eTable 1 Risk of bias assessment of 20 eligible randomized clinical trials

| **Author** | **D1** | **D2** | **D3** | **D4** | **D5** | **Overall** |
| --- | --- | --- | --- | --- | --- | --- |
| **All studies** | | | | | | |
| Fartoukh 2021 | Low | Low | Low | Low | Low | Low |
| Zhang XB 2022 | High | Some concerns | Low | Low | Low | High |
| Ding XW 2019 | Low | Some concerns | Low | Low | Low | Some concerns |
| Yuan GD 2020 | Low | Some concerns | Low | Low | Low | Some concerns |
| Cai ZY 2020 | Low | Some concerns | Low | Low | Low | Some concerns |
| Liu EQ 2013 | High | Some concerns | Low | Low | Low | High |
| Liu Y 2015 | High | Some concerns | Low | Low | Low | High |
| Gao DD 2023 | Low | Some concerns | Low | Low | Low | Some concerns |
| Tian YH 2014 | High | Some concerns | Low | Low | Low | High |
| Zhang L 2023 | Low | Some concerns | Low | Low | Low | Some concerns |
| Chen HS 2022 | Low | Some concerns | Low | Low | Low | Some concerns |
| Guan HX 2021 | Low | Some concerns | Low | Low | Low | Some concerns |
| Huang CC 2023 | High | Some concerns | Low | Low | Low | High |
| Zeng LH 2022 | Low | Some concerns | Low | Low | Low | Some concerns |
| Li HX 2021 | Low | Some concerns | Low | Low | Low | Some concerns |
| Li CY 2017 | Low | Some concerns | Low | Low | Low | Some concerns |
| Zou C 2024 | High | Some concerns | Low | Low | Low | High |
| Lin DX 2002 | High | Some concerns | Low | Low | Low | High |
| Zhang QY 2012 | High | Some concerns | Low | Low | Low | High |
| Xu YH 2019 | Low | Some concerns | Low | Low | Low | Some concerns |
| **Clinical efficacy** | | | | | | |
| Zhang XB 2022 | High | Some concerns | Low | Low | Low | High |
| Cai ZY 2020 | Low | Some concerns | Low | Low | Low | Some concerns |
| Liu EQ 2013 | High | Some concerns | Low | Low | Low | High |
| Gao DD 2023 | Low | Some concerns | Low | Low | Low | Some concerns |
| Tian YH 2014 | High | Some concerns | Low | Low | Low | High |
| Zhang L 2023 | Low | Some concerns | Low | Low | Low | Some concerns |
| Chen HS 2022 | Low | Some concerns | Low | Low | Low | Some concerns |
| Guan HX 2021 | Low | Some concerns | Low | Low | Low | Some concerns |
| Huang CC 2023 | High | Some concerns | Low | Low | Low | High |
| Li HX 2021 | Low | Some concerns | Low | Low | Low | Some concerns |
| Zeng LH 2022 | Low | Some concerns | Low | Low | Low | Some concerns |
| eTable 1 (continued) | | | | | | |
| Li CY 2017 | Low | Some concerns | Low | Low | Low | Some concerns |
| Zou C 2024 | High | Some concerns | Low | Low | Low | High |
| Lin DX 2002 | High | Some concerns | Low | Low | Low | High |
| Zhang QY 2012 | High | Some concerns | Low | Low | Low | High |
| Xu YH 2019 | Low | Some concerns | Low | Low | Low | Some concerns |
| Liu Y 2015 | High | Some concerns | Low | Low | Low | High |
| **Recurrence** | | | | | | |
| Liu EQ 2013 | High | Some concerns | Low | Low | Low | High |
| Liu Y 2015 | High | Some concerns | Low | Low | Low | High |
| Gao DD 2023 | Low | Some concerns | Low | Low | Low | Some concerns |
| Zhang L 2023 | Low | Some concerns | Low | Low | Low | Some concerns |
| Chen HS 2022 | Low | Some concerns | Low | Low | Low | Some concerns |
| Li CY 2017 | Low | Some concerns | Low | Low | Low | Some concerns |
| Zhang QY 2012 | High | Some concerns | Low | Low | Low | High |
| Xu YH 2019 | Low | Some concerns | Low | Low | Low | Some concerns |
| Fartoukh 2021 | Low | Some concerns | Low | Low | Low | Some concerns |
| **Mortality** | | | | | | |
| Liu EQ 2013 | High | Some concerns | Low | Low | Low | High |
| Liu Y 2015 | High | Some concerns | Low | Low | Low | High |
| Li CY 2017 | Low | Some concerns | Low | Low | Low | Some concerns |
| Xu YH 2019 | Low | Some concerns | Low | Low | Low | Some concerns |
| Fartoukh 2021 | Low | Low | Low | Low | Low | Low |
| **Overall complications** | | | | | | |
| Liu EQ 2013 | High | Some concerns | Low | Low | Low | High |
| Zhang L 2023 | Low | Some concerns | Low | Low | Low | Some concerns |
| Guan HX 2021 | Low | Some concerns | Low | Low | Low | Some concerns |
| Huang CC 2023 | High | Some concerns | Low | Low | Low | High |
| Zeng LH 2022 | Low | Some concerns | Low | Low | Low | Some concerns |
| Fartoukh 2021 | Low | Some concerns | Low | Low | Low | Some concerns |
| Zhang XB 2022 | High | Some concerns | Low | Low | Low | High |
| Zhang QY 2012 | High | Some concerns | Low | Low | Low | High |
| Tian YH 2014 | High | Some concerns | Low | Low | Low | High |
| Ding XW 2019 | Low | Some concerns | Low | Low | Low | Some concerns |
| Cai ZY 2020 | Low | Some concerns | Low | Low | Low | Some concerns |
| Li CY 2017 | Low | Some concerns | Low | Low | Low | Some concerns |
| eTable 1 (continued) | | | | | | |
| Xu YH 2019 | Low | Some concerns | Low | Low | Low | Some concerns |
| **Nausea/Vomiting** | | | | | | |
| Ding XW 2019 | Low | Some concerns | Low | Low | Low | Some concerns |
| Tian YH 2014 | High | Some concerns | Low | Low | Low | High |
| Guan HX 2021 | Low | Some concerns | Low | Low | Low | Some concerns |
| Li CY 2017 | Low | Some concerns | Low | Low | Low | Some concerns |
| Xu YH 2019 | Low | Some concerns | Low | Low | Low | Some concerns |
| **Fever** | | | | | | |
| Zhang XB 2022 | High | Some concerns | Low | Low | Low | High |
| Ding XW 2019 | Low | Some concerns | Low | Low | Low | Some concerns |
| Cai ZY 2020 | Low | Some concerns | Low | Low | Low | Some concerns |
| Zhang L 2023 | Low | Some concerns | Low | Low | Low | Some concerns |
| Guan HX 2021 | Low | Some concerns | Low | Low | Low | Some concerns |
| Huang CC 2023 | High | Some concerns | Low | Low | Low | High |
| Zhang QY 2012 | High | Some concerns | Low | Low | Low | High |
| **Chest pain** | | | | | | |
| Zhang XB 2022 | High | Some concerns | Low | Low | Low | High |
| Cai ZY 2020 | Low | Some concerns | Low | Low | Low | Some concerns |
| Zhang L 2023 | Low | Some concerns | Low | Low | Low | Some concerns |
| Guan HX 2021 | Low | Some concerns | Low | Low | Low | Some concerns |
| Huang CC 2023 | High | Some concerns | Low | Low | Low | High |
| Zeng LH 2022 | Low | Some concerns | Low | Low | Low | Some concerns |
| Li CY 2017 | Low | Some concerns | Low | Low | Low | Some concerns |
| Zhang QY 2012 | High | Some concerns | Low | Low | Low | High |
| Xu YH 2019 | Low | Some concerns | Low | Low | Low | Some concerns |
| **Abdominal pain** | | | | | | |
| Ding XW 2019 | Low | Some concerns | Low | Low | Low | Some concerns |
| Li CY 2017 | Low | Some concerns | Low | Low | Low | Some concerns |
| Xu YH 2019 | Low | Some concerns | Low | Low | Low | Some concerns |
| **Cough** | | | | | | |
| Li CY 2017 | Low | Some concerns | Low | Low | Low | Some concerns |
| Zhang QY 2012 | High | Some concerns | Low | Low | Low | High |
| Xu YH 2019 | Low | Some concerns | Low | Low | Low | Some concerns |
| **Palpitation** | | | | | | |
| Cai ZY 2020 | Low | Some concerns | Low | Low | Low | Some concerns |
| eTable 1 (continued) | | | | | | |
| Huang CC 2023 | High | Some concerns | Low | Low | Low | High |
| Li CY 2017 | Low | Some concerns | Low | Low | Low | Some concerns |
| Xu YH 2019 | Low | Some concerns | Low | Low | Low | Some concerns |
| **Chest distress** | | | | | | |
| Tian YH 2014 | High | Some concerns | Low | Low | Low | High |
| Zeng LH 2022 | Low | Some concerns | Low | Low | Low | Some concerns |
| **Time to hemostasis** | | | | | | |
| Zhang XB 2022 | High | Some concerns | Low | Low | Low | High |
| Cai ZY 2020 | Low | Some concerns | Low | Low | Low | Some concerns |
| Zhang L 2023 | Low | Some concerns | Low | Low | Low | Some concerns |
| Huang CC 2023 | High | Some concerns | Low | Low | Low | High |
| Zhang QY 2012 | High | Some concerns | Low | Low | Low | High |
| **FEV1/FVC** | | | | | | |
| Zhang XB 2022 | High | Some concerns | Low | Low | Low | High |
| Yuan GD 2020 | Low | Some concerns | Low | Low | Low | Some concerns |
| Gao DD 2023 | Low | Some concerns | Low | Low | Low | Some concerns |
| Li CY 2017 | Low | Some concerns | Low | Low | Low | Some concerns |
| Xu YH 2019 | Low | Some concerns | Low | Low | Low | Some concerns |
| **PaO2** | | | | | | |
| Yuan GD 2020 | Low | Some concerns | Low | Low | Low | Some concerns |
| Gao DD 2023 | Low | Some concerns | Low | Low | Low | Some concerns |
| Li CY 2017 | Low | Some concerns | Low | Low | Low | Some concerns |
| Xu YH 2019 | Low | Some concerns | Low | Low | Low | Some concerns |
| **PaCO2** | | | | | | |
| Yuan GD 2020 | Low | Some concerns | Low | Low | Low | Some concerns |
| Gao DD 2023 | Low | Some concerns | Low | Low | Low | Some concerns |
| Li CY 2017 | Low | Some concerns | Low | Low | Low | Some concerns |
| Xu YH 2019 | Low | Some concerns | Low | Low | Low | Some concerns |
| **PH** | | | | | | |
| Yuan GD 2020 | Low | Some concerns | Low | Low | Low | Some concerns |
| Gao DD 2023 | Low | Some concerns | Low | Low | Low | Some concerns |
| Li CY 2017 | Low | Some concerns | Low | Low | Low | Some concerns |
| Xu YH 2019 | Low | Some concerns | Low | Low | Low | Some concerns |

D1: Risk of bias arising from the randomization process; D2: Risk of bias due to deviations from the intended interventions; D3: Risk of bias due to missing outcome data; D4: Risk of bias in measurement of the outcome; D5: Risk of bias in selection of the reported result; Overall: Overall risk of bias

# eTable 2 Subgroup analyses for clinical efficacy, recurrence, and mortality

| Analysis | | Clinical efficacy | | | Recurrence | | | Mortality | | |
| --- | --- | --- | --- | --- | --- | --- | --- | --- | --- | --- |
|  |  | No. studies | RR  (95%CI) | Test of interaction p-value | No. studies | RR  (95%CI) | Test of interaction p-value | No. studies | RR  (95%CI) | Test of interaction p-value |
| Subgroup analysis by follow-up time | 1 Month | 12 | 1.19(1.10-1.29) | 0.42 | 3 | 0.80(0.07-9.25) | 0.84 | 3 | 0.36(0.05-2.43) | 0.55 |
|  | 3 Months | 2 | 1.33(1.08-1.63) |  | 1 | 0.31(0.13-0.75) |  | 0 | - |  |
|  | 6 Months | 3 | 1.30(1.14-1.48) |  | 6 | 0.33(0.19-0.57) |  | 3 | 0.45(0.22-0.95) |  |
|  | 12 Months | 0 | - |  | 2 | 0.20(0.04-1.11) |  | 0 | - |  |

RR: relative risk; 95%CI: 95% confidence interval;

NA: not applicable due to less than 2 studies in a given subgroup.

# eTable 3 Subgroup analyses for overall complications, nausea/vomiting, fever, and chest pain

| Analysis | | Overall complications | | | Nausea/Vomiting | | | Fever | | | Chest pain | | |
| --- | --- | --- | --- | --- | --- | --- | --- | --- | --- | --- | --- | --- | --- |
|  |  | No. studies | RR  (95%CI) | Test of interaction p-value | No. studies | RR  (95%CI) | Test of interaction p-value | No. studies | RR  (95%CI) | Test of interaction p-value | No. studies | RR  (95%CI) | Test of interaction p-value |
| Subgroup analysis by follow-up time | 1 Month | 8 | 0.59(0.38-0.93) | 0.72 | 3 | 0.10(0.02-0.52) | 0.86 | 6 | 0.99(0.35-2.80) | 0.16 | 5 | 0.53(0.14-1.95) | 0.59 |
|  | 3 Months | 4 | 0.76(0.26-2.27) |  | 2 | 0.13(0.02-0.98) |  | - | - |  | 3 | 0.73(0.20-2.62) |  |
|  | 6 Months | 1 | 0.88(0.36-2.11) |  | - | - |  | 1 | 9.00(0.51-160.17) |  | 1 | 0.13(0.01-2.39) |  |

RR: relative risk; 95%CI: 95% confidence interval;

NA: not applicable due to less than 2 studies in a given subgroup.

eTable 4 GRADE evidence profile of adverse events

| Outcome | No. of trials  (No. of patients) | Risk of bias | Inconsistency | Indirectness | Imprecision | Publication bias | Treatment association (95% CI) | | Overall certainty of evidence |
| --- | --- | --- | --- | --- | --- | --- | --- | --- | --- |
|  |  |  |  |  |  |  | BAE | Control group |  |
| Nausea/Vomiting | 5(348) | No(20.26%) | I^2^=0% | No | Yes | NA | 0 (0%) | 21 (12%) | Moderate |
|  |  |  |  |  |  |  | ARR 10.74% (7.24% to 11.71%) | |  |
|  |  |  |  |  |  |  | RR 0.11 (0.03 to 0.40) | |  |
| Fever | 7(482) | No(49.44%) | I^2^=0% | No | Yes | NA | 12 (5%) | 8 (3%) | Moderate |
|  |  |  |  |  |  |  | ARR -0.93% (-7.97% to1.73 %) | |  |
|  |  |  |  |  |  |  | RR 1.28 (0.48 to 3.40) | |  |
| Chest pain | 9(641) | No(32.17%) | I^2^=0% | No | Yes | NA | 9 (3%) | 17 (5%) | Moderate |
|  |  |  |  |  |  |  | ARR 2.45% (-1.23% to 4.10%) | |  |
|  |  |  |  |  |  |  | RR 0.54 (0.23 to 1.23) | |  |
| Abdominal pain | 3(200) | No(0%) | I^2^=0% | No | Yes | NA | 0 (0%) | 4 (4%) | Moderate |
|  |  |  |  |  |  |  | ARR 2.88% (-2.68% to 3.8%) | |  |
|  |  |  |  |  |  |  | RR 0.28 (0.05 to 1.67) | |  |

eTable 4 (continued)

| Outcome | No. of trials  (No. of patients) | Risk of bias | Inconsistency | Indirectness | Imprecision | Publication bias | Treatment association (95% CI) | | Overall certainty of evidence |
| --- | --- | --- | --- | --- | --- | --- | --- | --- | --- |
|  |  |  |  |  |  |  | BAE | Control group |  |
| Cough | 3(200) | No(34.82%) | I^2^=63.19% ↓ | No | Yes | NA | 3 (3%) | 8 (8%) | Low |
|  |  |  |  |  |  |  | ARR 0.8% (-116.08% to 7.6%) | |  |
|  |  |  |  |  |  |  | RR 0.90 (0.05 to 15.51) | |  |
| Palpitation | 4(304) | No(27.22%) | I^2^=0% | No | Yes | NA | 4 (3%) | 7 (5%) | Moderate |
|  |  |  |  |  |  |  | ARR 1.75% (-5.30% to 3.78%) | |  |
|  |  |  |  |  |  |  | RR 0.62 (0.18 to 2.15) | |  |
| Chest distress | 2(170) | Yes(70.02%) | I^2^=0% | No | Yes | NA | 5 (6%) | 4 (5%) | Low |
|  |  |  |  |  |  |  | ARR -1.08% (-16.39% to 3.11%) | |  |
|  |  |  |  |  |  |  | RR 1.23 (0.34 to 4.48) | |  |

BAE:Bronchial artery embolization; Control group:including pharmacotherapy, surgery, usual care and bronchoscopic balloon tamponade;RR: relative risk; ARR: absolute risk reduction;

CI: 95% confidence interval; NA: not applicable due to less than 10 studies; ↓:downgrade by one level .

eTable 5 GRADE evidence profile of time to hemostasis, FEV1/FVC, PaO2, PaCO2, and PH

| Outcome | No. of trials  (No. of patients) | Risk of bias | Inconsistency | Indirectness | Imprecision | Publication bias | Treatment association  (95% CI) | Overall certainty of evidence |
| --- | --- | --- | --- | --- | --- | --- | --- | --- |
|  |  |  |  |  |  |  |  |  |
|  |  |  |  |  |  |  |  |  |
|  |  |  |  |  |  |  |  |  |
|  |  |  |  |  |  |  |  |  |
| Time to hemostasis (day) | 5(362) | Yes(60.16%) | I^2^=99.42%↓ | No | Yes | NA | WMD -0.54 day  (95%CI -1.84 to +0.76 day) | Very Low |
| FEV_1_/FVC(%) | 5(415) | No(27.86%) | I^2^=83.37%↓ | No | Yes | NA | WMD 7.62  (95%CI 4.80 to 10.45) | Low |
| PaO_2_ | 4(337) | No(0%) | I^2^=99.75%↓ | No | Yes | NA | WMD 1.38  (95%CI 0.48 to +2.28 ) | Low |
| PaCO_2_ | 4(337) | No(0%) | I^2^=99.66%↓ | No | Yes | NA | WMD -1.00  (95%CI -1.68 to -0.32 ) | Low |
| PH | 4(337) | No(0%) | I^2^=99.66%↓ | No | Yes | NA | WMD 0.11  (95%CI -0.06 to +0.29) | Low |

BAE:Bronchial artery embolization; WMD: weighted mean difference; CI: 95% confidence interval;

NA: not applicable due to less than 10 studies; ↓:downgrade by one level .

eTable 6 Sensitivity analysis for binary outcomes

| Outcomes | Sensitivity Analysis | No. of studies | Total events(n) | Total sample size (N) | RR | 95% CI | |
| --- | --- | --- | --- | --- | --- | --- | --- |
| Clinical efficacy | Primary Analysis (D-L method) | 17 | 1145 | 1345 | 1.22 | 1.14 | 1.30 |
|  | HKSJ method | 17 | 1145 | 1345 | 1.23 | 1.14 | 1.32 |
| Recurrence | Primary Analysis (D-L method) | 9 | 105 | 616 | 0.38 | 0.21 | 0.69 |
|  | HKSJ method | 9 | 105 | 616 | 0.39 | 0.18 | 0.86 |
| Mortality | Primary Analysis (D-L method) | 5 | 15 | 356 | 0.25 | 0.08 | 0.81 |
|  | HKSJ method | 5 | 15 | 356 | 0.25 | 0.08 | 0.84 |
| Overall complications | Primary Analysis (D-L method) | 13 | 225 | 924 | 0.63 | 0.44 | 0.91 |
|  | HKSJ method | 13 | 225 | 924 | 0.63 | 0.37 | 1.07 |
| Nausea/Vomiting | Primary Analysis (D-L method) | 5 | 21 | 348 | 0.11 | 0.03 | 0.40 |
|  | HKSJ method | 5 | 21 | 348 | 0.11 | 0.03 | 0.40 |
| Fever | Primary Analysis (D-L method) | 7 | 20 | 482 | 1.28 | 0.48 | 3.40 |
|  | HKSJ method | 7 | 20 | 482 | 1.38 | 0.43 | 4.47 |
| Chest pain | Primary Analysis (D-L method) | 9 | 26 | 641 | 0.54 | 0.23 | 1.23 |
|  | HKSJ method | 9 | 26 | 641 | 0.56 | 0.20 | 1.55 |
| Abdominal pain | Primary Analysis (D-L method) | 3 | 4 | 200 | 0.28 | 0.05 | 1.67 |
|  | HKSJ method | 3 | 4 | 200 | 0.28 | 0.05 | 1.67 |
| Cough | Primary Analysis (D-L method) | 3 | 11 | 200 | 0.90 | 0.05 | 15.51 |
|  | HKSJ method | 3 | 11 | 200 | 0.90 | 0.05 | 14.88 |
| Palpitation | Primary Analysis (D-L method) | 4 | 11 | 304 | 0.62 | 0.18 | 2.15 |
|  | HKSJ method | 4 | 11 | 304 | 0.62 | 0.16 | 2.34 |
| Chest distress | Primary Analysis (D-L method) | 2 | 9 | 170 | 1.23 | 0.34 | 4.48 |
|  | HKSJ method | 2 | 9 | 170 | 1.23 | 0.33 | 4.60 |

eTable 7 Sensitivity analysis for continuous outcomes

| **Outcomes** | **Sensitivity Analysis** | **No. of studies** | **Total sample size (N)** | **WMD** | **95%CI** | |
| --- | --- | --- | --- | --- | --- | --- |
| Time to hemostasis | Primary Analysis (D-L method) | 5 | 362 | -0.54 | -1.84 | 0.76 |
|  | HKSJ method | 5 | 362 | -0.51 | -3.00 | 1.98 |
| FEV_1_/FVC | Primary Analysis (D-L method) | 5 | 415 | 7.62 | 4.80 | 10.45 |
|  | HKSJ method | 5 | 415 | 7.49 | 4.24 | 10.74 |
| PaO_2_ | Primary Analysis (D-L method) | 4 | 337 | 1.38 | 0.48 | 2.28 |
|  | HKSJ method | 4 | 337 | 2.61 | -1.54 | 6.77 |
| PaCO_2_ | Primary Analysis (D-L method) | 4 | 337 | -1.00 | -1.68 | -0.32 |
|  | HKSJ method | 4 | 337 | -2.03 | -5.18 | 1.11 |
| PH | Primary Analysis (D-L method) | 4 | 337 | 0.11 | -0.06 | 0.29 |
|  | HKSJ method | 4 | 337 | 0.12 | -0.04 | 0.27 |

eTable 8 Journal Information and Impact Factors of the 19 Chinese References

| **No.** | **Study (first author)** | **Journal** | **Publication type** | **Peer-review status** | **Chinese impact factor (non-SCI)** |
| --- | --- | --- | --- | --- | --- |
| 24 | Liu et al. (刘恩强) | 现代诊断与治疗 | Journal article | Reported peer-reviewed | 0.73–0.87 (CNKI composite, non-SCI) |
| 25 | Chen et al. (陈洪胜) | 医学理论与实践 | Journal article | Peer-reviewed | 0.51 (CNKI composite, non-SCI) |
| 26 | Zeng et al. (曾兰辉) | 中国医学创新 | Journal article | Peer-reviewed | 0.58–1.31 (CNKI composite, non-SCI) |
| 27 | Huang et al. (黄晨晨) | 中外医疗 | Journal article | Reported peer-reviewed | 0.25–0.78 (CNKI composite, non-SCI) |
| 28 | Zhang et al. (张秀彬) | 甘肃科技纵横 | Journal article | Peer-reviewed | 0.33–0.74 (CNKI composite, non-SCI) |
| 29 | Zhang et al. (张倩云) | 中国医疗前沿 | Journal article | Reported peer-reviewed | 0.44 (CNKI composite, non-SCI) |
| 30 | Guan et al. (管恒星) | 当代医药论丛 | Journal article | Reported peer-reviewed | 0.91 (CNKI composite, non-SCI) |
| 31 | Zhang et al. (张蕾) | 航空航天医学杂志 | Journal article | Reported peer-reviewed | 0.98–1.48 (CNKI composite, non-SCI) |
| 32 | Gao et al. (高冬冬) | 大医生 | Journal article | Peer-reviewed | 0.65 (CNKI composite, non-SCI) |
| 33 | Tian et al. (田玉恒) | 中国实用医药 | Journal article | Reported peer-reviewed | 0.92–1.32 (CNKI composite, non-SCI) |
| 34 | Ding et al. (丁秀文) | 临床医药文献电子杂志 | Journal article | Reported peer-reviewed | 0.27–0.81 (CNKI composite, non-SCI) |
| 35 | Cai et al. (蔡宗洋) | 临床医学工程 | Journal article | Reported peer-reviewed | 0.42–1.42 (CNKI composite, non-SCI) |
| 36 | Yuan et al. (袁艮弟) | 现代医学与健康研究电子杂志 | Journal article | Peer-reviewed | 0.27–0.73 (CNKI composite, non-SCI) |
| 37 | Lin et al. (林丹曦) | 中华急诊医学杂志 | Journal article | Peer-reviewed | 1.30–2.21 (CNKI composite, non-SCI); 核心IF 1.63 (ISTIC, non-SCI) |
| 38 | Li et al. (李洪晓) | 家有孕宝 | Journal article | Reported peer-reviewed | Not available / 0.00 (non-SCI) |
| 39 | Zou et al. (邹晨) | 中国实用医药 | Journal article | Reported peer-reviewed | 0.92–1.32 (CNKI composite, non-SCI) |
| 40 | Li et al. (李成业) | 广西医科大学学报 | Journal article | Peer-reviewed | 1.17–2.39 (CNKI composite, non-SCI); 核心IF 1.34 (ISTIC, non-SCI) |
| 41 | Liu et al. (刘扬) | 中国微生态学杂志 | Journal article | Peer-reviewed | 1.54 (CNKI composite, non-SCI); 核心IF 1.74 (ISTIC, non-SCI) |
| 42 | Xu et al. (许雅) | 医学理论与实践 | Journal article | Peer-reviewed | 0.51 (CNKI composite, non-SCI) |

Peer-review status was re-evaluated using official journal websites and publicly available journal information. “Peer-reviewed” indicates that an explicit peer-review or external expert-review procedure could be independently verified. “Reported peer-reviewed” indicates that peer-review status was reported by the publication source, but specific publicly available documentation could not be independently identified.

**Notes**

Important clarification on impact factors:

1. JCR impact factor (Journal Citation Reports impact factor) is produced by Clarivate/Web of Science for journals indexed in SCIE/SSCI.

2. The 19 journals listed in this table are all Chinese-language medical journals. As of the latest available data, NONE of them are indexed in SCIE, therefore they do NOT have an official JCR impact factor.

3. The values in the column 'Chinese impact factor (non-SCI)' come from Chinese domestic evaluation systems, mainly:

- CNKI 'China Academic Journal Impact Factor Yearbook' (复合影响因子 / 综合影响因子)

- ISTIC 'Chinese Science and Technology Journal Citation Report' (中信所核心影响因子)

4. These Chinese impact factors are NOT equivalent to SCI/JCR impact factors. They use different databases, different citation windows, and different calculation rules. They should be reported separately and not compared directly with JCR IF values.

Sources consulted: CNKI, Wanfang, VIP, journal official websites, Baidu Baike, and third-party journal evaluation platform

eTable 9 Qualitative justification for GRADE risk-of-bias judgments for the main outcomes

| **No.** | **Outcome** | **Treatment contrast** | **High-RoB weight** | **Main concerns** | **Primary estimate** | **After excluding High-RoB trials** | **RoB judgment** | **Justification** |
| --- | --- | --- | --- | --- | --- | --- | --- | --- |
| 1 | Clinical efficacy | BAE vs pharmacotherapy | 55.22% | Randomization/allocation concealment | RR 1.24 (1.18–1.31) | RR 1.27 (1.18–1.37), 7 trials | Not serious | Effect direction and magnitude remained similar after exclusion of High-RoB trials. |
| 2 | Clinical efficacy | BAE + usual care vs usual care | 74.25% | Randomization/allocation concealment; limited lower-risk evidence | RR 1.27 (1.12–1.43) | RR 1.27 (1.01–1.61), 1 trial | Serious ↓ | High-RoB trials dominated; only one lower-risk trial remained. |
| 3 | Clinical efficacy | BAE vs bronchoscopic balloon tamponade | 37.71% | Randomization/allocation concealment | RR 0.91 (0.80–1.03) | RR 0.90 (0.77–1.05), 1 trial | Not serious | High-RoB evidence did not dominate and the effect estimate remained similar. |
| 4 | Recurrence | BAE vs pharmacotherapy | 0% | Some concerns regarding randomization | RR 0.26 (0.13–0.54) | NA | Not serious | No contributing trial was judged at overall High RoB. |
| 5 | Recurrence | BAE + usual care vs usual care | 45.65% | Randomization/allocation concealment | RR 0.28 (0.15–0.53) | RR 0.31 (0.13–0.75), 1 trial | Not serious | High-RoB evidence did not dominate; the remaining estimate was similar in direction and magnitude. |
| 6 | Recurrence | BAE vs bronchoscopic balloon tamponade | 55.13% | Randomization/allocation concealment; limited lower-risk evidence | RR 2.14 (0.17–27.10) | RR 9.00 (1.21–66.70), 1 trial | Serious ↓ | Exclusion of the High-RoB trial materially changed the estimate and left one lower-risk trial. |
| 7 | Mortality | BAE vs pharmacotherapy | 0% | Some concerns regarding randomization | RR 0.20 (0.05–0.90) | NA | Not serious | No contributing trial was judged at overall High RoB. |
| 8 | Mortality | BAE + usual care vs usual care | 76.15% | Randomization/allocation concealment; limited lower-risk evidence | RR 0.36 (0.05–2.43) | RR 1.11 (0.02–54.68), 1 trial | Serious ↓ | High-RoB trials dominated and exclusion markedly changed the point estimate. |
| 9 | Overall complications | BAE vs pharmacotherapy | 40.54% | Randomization/allocation concealment | RR 0.41 (0.26–0.63) | RR 0.43 (0.24–0.76), 5 trials | Not serious | Exclusion of High-RoB trials yielded a very similar estimate. |
| 10 | Overall complications | BAE + usual care vs usual care | 60.29% | Randomization/allocation concealment; limited lower-risk evidence | RR 1.04 (0.45–2.39) | RR 2.23 (0.34–14.84), 2 trials | Serious ↓ | High-RoB evidence contributed most of the weight and exclusion materially changed the point estimate. |
| 11 | Overall complications | BAE vs bronchoscopic balloon tamponade | 66.98% | Randomization/allocation concealment; limited lower-risk evidence | RR 1.15 (0.54–2.46) | RR 2.00 (0.55–7.27), 1 trial | Serious ↓ | High-RoB evidence dominated; exclusion changed the estimate and left one lower-risk trial. |

BAE,:bronchial artery embolization; CI: confidence interval; RR: risk ratio; RoB: risk of bias; NA, not applicable.

**Notes:** High-RoB weight represents the proportion of meta-analytic weight contributed by trials judged to be at overall high risk of bias within each treatment contrast. Sensitivity analyses excluded trials judged to be at overall high risk of bias. When only one trial remained, the estimate from that trial is shown without further pooling.

eFig. 1 Forest plot for clinical efficacy

eFig. 2 Forest plot for time to hemostasis

eFig. 3 Forest plot for recurrence

eFig. 4 Forest plot for overall complications

eFig. 5 Comparator-specific analysis of mortality

eFig. 6 Forest plot for Nausea/Vomiting

eFig. 7 Forest plot for chest pain

eFig. 8 Forest plot for fever

eFig. 9 Forest plot for FEV1/FVC

eFig. 10 Sensitivity analysis excluding trials at high risk of bias for clinical efficacy

eFig. 11 Sensitivity analysis excluding trials at high risk of bias for recurrence

eFig. 12 Sensitivity analysis excluding trials at high risk of bias for mortality

eFig. 13 Sensitivity analysis excluding trials at high risk of bias for overall complications

eFig.14 Funnel plot for clinical efficacy (BAE vs pharmacotherapy)

eAppendix 1: Search Strategies

PubMed:

((Interventional therapy[Title/Abstract]) OR (Bronchial artery embolization[Title/Abstract]) OR (Bronchial artery embolisation[Title/Abstract]) OR (BAE[Title/Abstract]) OR (transcatheter embolization[Title/Abstract]) OR (TAE[Title/Abstract])) AND (("Hemoptysis"[Mesh]) OR (haemoptysis[Title/Abstract]) OR (hemoptysis[Title/Abstract]) OR (Hemoptyses[Title/Abstract]))

Embase:

('interventional therapy':ab,ti OR 'bronchial artery embolization':ab,ti OR 'bronchial artery embolisation':ab,ti OR bae:ab,ti OR 'transcatheter embolization':ab,ti OR tae:ab,ti) AND ('hemoptysis':ab,ti OR haemoptysis:ab,ti OR hemoptyses:ab,ti OR haemoptyses:ab,ti OR emptysis:ab,ti)

Cochrane:

Cochrane:((Interventional therapy):ti,ab,kw OR (BAE):ti,ab,kw OR ("bronchial artery embolization"):ti,ab,kw OR (transcatheter embolization):ti,ab,kw OR (TAE):ti,ab,kw) AND (("hemoptysis"):ti,ab,kw OR ("hemoptyses"):ti,ab,kw)

China Science and Technology Journal Database (CQVIP):

(((((任意字段=咯血 OR 任意字段=emptysis) OR 任意字段=haemoptysis) OR 任意字段=hemoptysis) OR 任意字段=Hemoptyses) AND (任意字段=支气管动脉栓塞术 OR 任意字段=BAE))

Wanfang Database:

主题:(咯血 or emptysis or haemoptysis or hemoptysis or Hemoptyses)and主题:(支气管动脉栓塞术 or BAE)

Sinomed:

("咯血"[常用字段:智能] OR "emptysis"[常用字段:智能] OR "haemoptysis"[常用字段:智能] OR "hemoptysis"[常用字段:智能] OR "Hemoptyses"[常用字段:智能]) AND ("支气管动脉栓塞术"[常用字段:智能] OR "BAE"[常用字段:智能])

China National Knowledge Infrastructure (CNKI):

（主题：咯血 + emptysis + haemoptysis + hemoptysis + Hemoptyses）AND（主题：支气管动脉栓塞术+BAE）
